# Training on multiple days results in better learning in embedded eyeblink conditioning in young human adults

**DOI:** 10.1038/s41539-025-00347-w

**Published:** 2025-08-14

**Authors:** Robert Winton, Meenam Pious, Anders Rasmussen

**Affiliations:** Associative Learning Lab, Department of Experimental Medical Science, Faculty of Medicine, Lund University, Lund, Sweden

**Keywords:** Cognitive neuroscience, Learning and memory, Human behaviour

## Abstract

Eyeblink conditioning is mediated by similar cerebellar pathways in humans and animals and is typically investigated using delay or trace protocols. These studies show that humans can easily acquire eyeblink conditioning within a single day of training whereas animals usually require around 3–10 days of acquisition training before they consistently exhibit conditioned responses. We aimed to study how a multiple-day acquisition training, across 3 non-consecutive days of a month, with 100 trials per day affected learning in young human adults. We employed an embedded protocol in which the US is embedded within the CS duration without co-termination. Our findings show, for the first time in humans using this protocol, that learning improves substantially on days 2 and 3. Our findings encourage research into how human cerebellum mediates consolidation across several days of eyeblink conditioning as well as into the neurocognitive mechanisms of the relatively underexplored embedded eyeblink conditioning protocol.

## Introduction

In Pavlovian^1^ or classical conditioning, a neutral, innocuous stimulus called a conditional stimulus (CS) is repeatedly paired with an unconditional stimulus (US) that elicits an unconditional response (UR). Gradually, the presentation of the CS alone can elicit a similar response prior to the onset of the expected US. This learned response is called a conditioned response (CR)^2^. Classical conditioning has been extensively employed to investigate the behavioral and neuronal mechanisms of learning, memory and cerebellar function^3^. This study aimed to examine how a simple form of classical conditioning, called eyeblink conditioning, changes over several days of training in human beings.

Eyeblink conditioning relies primarily on the cerebellum^4^. The US can be corneal air puff or a periorbital electric shock. This stimulus is mediated to the cerebellum via climbing fibers originating in inferior olive. This US naturally elicits an unconditioned eyeblink response (UR). The CS can be peripheral stimulations such as a tone or a light. This stimulus is mediated to the cerebellum via mossy fibers originating in pons^5^. With repeated CS-US pairings, Purkinje cells in the cerebellar cortex acquire a pause response that disinhibit cerebellar nuclei cells that trigger the CR. Learning in eyeblink conditioning is seen as an increase in the percentage of CRs, an increase in CR amplitude, or a shift in CR onset^2,3,6,7^. The emergence of valid conditioned eyeblink responses, usually measured as a change in the percentage of CRs^8–10^, can be used to indicate whether learning has happened or not.

Two often used conditioning models are delay and trace eyeblink conditioning. In delay eyeblink conditioning, the CS overlaps and co-terminates with the US, while there is a stimulus-free trace interval between CS and US in trace conditioning^11^. Importantly, the cerebellar network is sufficient for delay eyeblink conditioning. However, the addition of a stimulus-free interval between the CS and the US in trace eyeblink conditioning causes an additional reliance on the hippocampal-forebrain circuitry to potentiate that trace interval^2,3,6,7,11,12^. In trace conditioning a small temporal gap separates CS offset and US onset, and the CS offset in trace conditioning is suspected to serve as an indicator to initiate CR in human adults^13^. This speculation emerged from observing an extra spike 450 ms prior to the US onset in trace condition but not in the long-delay condition. A less explored variant is called an embedded protocol during which the US is embedded within the duration of the CS^14,15^. Thus, during an embedded protocol, the CS and the US overlap without co-terminating. Though similar to a delay paradigm, an advantage of letting the CS continue beyond the US is that learned responses can be attributed to the anticipated onset of the US rather than the cessation of the CS. An appetitive conditioning study in rats using embedded protocol examined the necessity of positive contingency for successful conditioning^16^. It was observed that a single embedded CS was sufficient for successful conditioning even during zero and negative contingency conditions, and that positive contingency is not a necessary condition. While studying the effect of intertrial USs in appetitive conditioning in rats, it was observed that the intertrial US had greater interfering effects on trace^17^ and delay^14,17^ conditioned rats than embedded conditioned rats. However, only few eyeblink conditioning studies employed an embedded protocol, and thus not much is known about the neurocognitive mechanisms underlying embedded eyeblink conditioning.

Studies have shown that the general mechanisms underlying eyeblink conditioning are similar across certain species, such as rats and rabbits^18^. However, there are also notable differences in the learning rates between different species. The acquisition or the emergence of eyeblink CRs can take 4–10 days in mice^9,19,20^, around 5 days in rats^21,22^, and 10–15 days in rabbits^23–25^. In humans, delay eyeblink CRs can be acquired within 30–150 trials in a single day^7,12,26^. It is not clear to date what mechanisms underlie this difference in the pace of CR acquisition between humans and animals, and there remains a dearth of studies investigating eyeblink conditioning in humans over extended periods. Here, we aimed to answer the following question: How does a multiple day eyeblink conditioning with 300 cumulative trials influence CR acquisition in young human adults? Unlike in previous studies, we used an embedded protocol to ensure that learned responses are attributable to the US onset. We hypothesized that the extended exposure across three days would lead to significant improvements in learning.

## Results

**Fig. 1:**
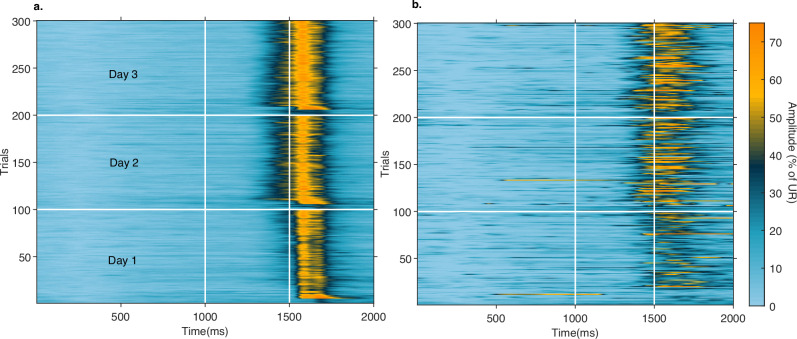
Heatplots on blink amplitude on successive training days. **a** Shows the amplitude on all trials (paired and CS-alone) before, during and after the presentation of the tone and the airpuff (vertical white lines) on three training days (horizontal white lines). **b** Shows the blink amplitude on CS-alone trials where there was no airpuff. CRs emerged on day one (trials 0–100) and became more frequent with larger amplitudes on days two and three (trials 101–300).

**Fig. 2:**
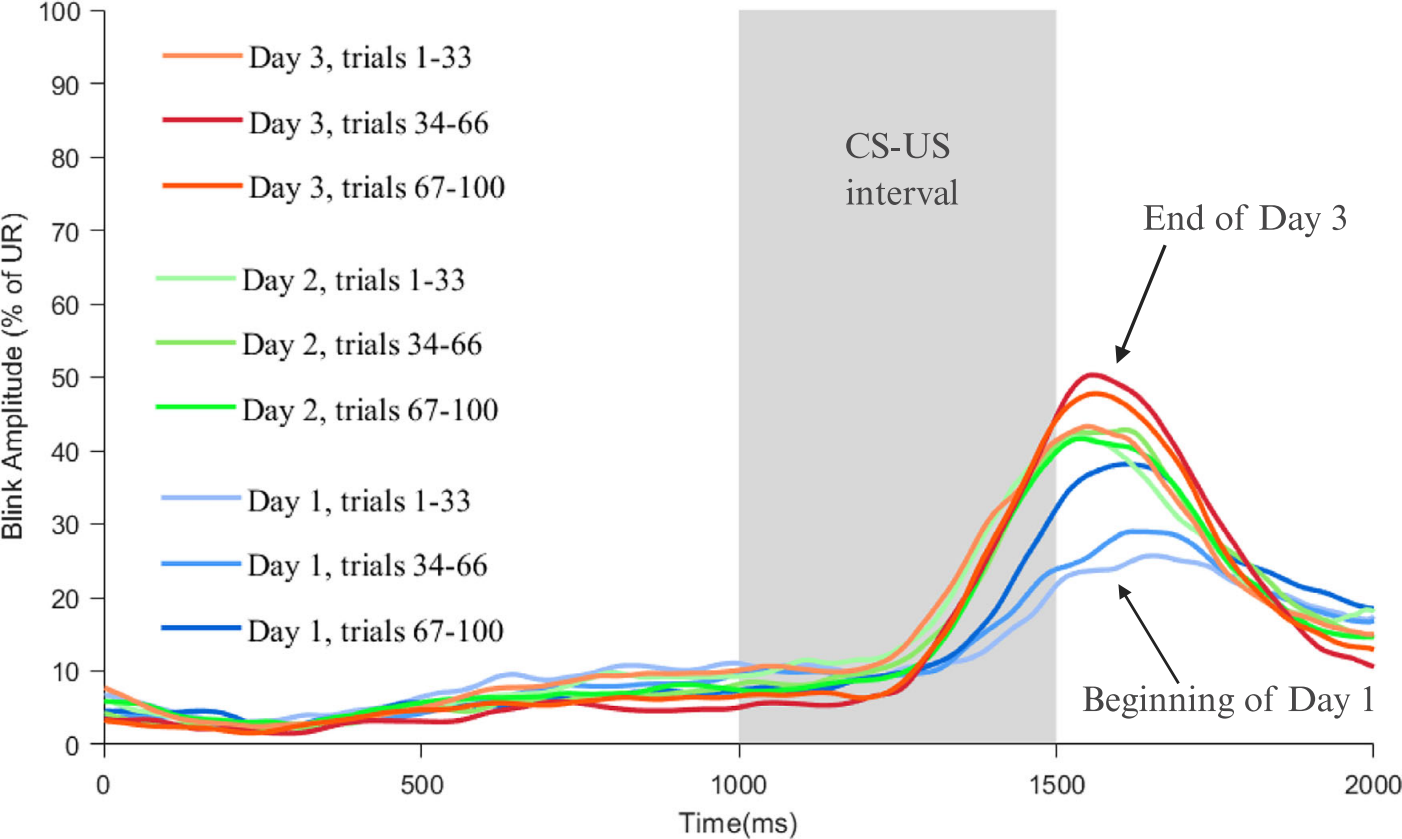
Average blink profiles on successive training days. Average blink profiles on CS-alone trials on three days of training, showing marked improvement in blink amplitude by the end of day 3.

The increase in CR amplitude across 3 days of training is illustrated in Figs. 1 and 2. It can also be seen that the CR frequency and CR percentage increase on the second and third training days suggesting that the learning continued to improve.

### Increase in CR percentage across 3 days

**Fig. 3:**
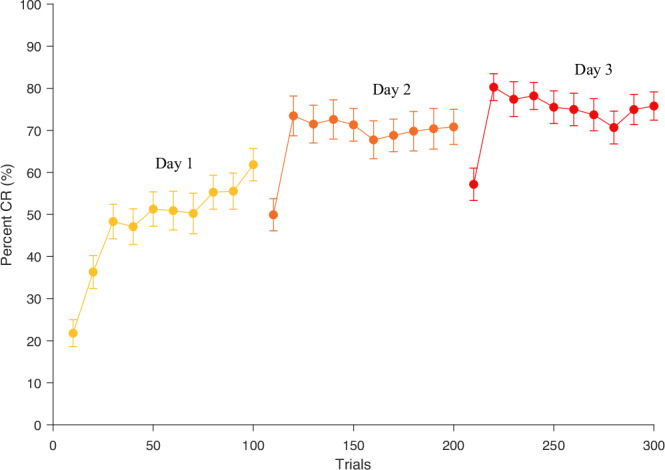
CR percentage on successive training days. Shows the CR percentage (mean + /−SEM) on successive blocks of 10 trials across the three training days.

To test whether such an extended training has a significant effect on learning, we employed a linear mixed effects model to test the effects of day on the CR percentage. We used linear mixed effects model because of its higher statistical power compared to repeated measures ANOVA and the ability to account for random variability, individual differences and missing data points in longitudinal datasets^27^^,^^28^. As fixed effects, we used day (1,2,3) and sex (male/female). As a random effect, we used the participant ID. The model was a random intercept model with a fixed slope implemented in Matlab R2018b, with the built-in function ‘fitlme’ using the formula *(‘CR percent~ Day + Sex* + *(1|Subject ID) ‘)*. The model showed that day has a significant effect on the CR percentage (*t* = 12.106, *p* = 7.3175e-52, CI = 0.078 0.108). For each day, the CR percentage increases by 9.3%. This translates as a 28% increase in CR percentage over 3 days. To further understand how the CR percentages varied between days 1 and 2 and between days 2 and 3, we employed two additional linear mixed effects models. On day 2, the CR percentage significantly increased by 14.4%, compared to day 1 (*t* = 9.41 *p* = 6.691e-20 CI = 0.114, 0.175). On day 3, the CR percentage significantly increased by 5.2%, compared to day 2 (*t* = 3.96 *p* = 8.1616e-05 CI = 0.026 0.078). Figure 3 shows the increase in CR percentage across all 3 training days. We observed that eyeblink CRs gradually emerged on day 1, and our results demonstrate that production of eyeblink CRs continued to improve with training on days 2 and 3. This total increase in learning, accumulated over the 300 trials, highlights the importance of both the number of days and the total number of trials in enhancing cerebellar learning and response acquisition.

### Intervals between training days

The intervals between training days were not fixed and varied between participants. To understand whether this variation between the training sessions affected the CR percentage, we divided the participant data into two groups: Group A (*n* = 26) had participants with the training intervals longer than 5 days, and group B (*n* = 19) consisted of those with training intervals of 5 days or less. A linear mixed effects model was used to analyse the effect of training interval as a predictor. The results indicated no significant difference in CR percentage between the two groups (*t* = −1.13, *p* = 0.255, CI = −0.18, 0.047).

### Variation in CR onset latency

Additionally, we tested if variation in CR onset latency was affected by the day of training. For that purpose, we employed a separate linear mixed effects model using the formula *(‘CR onset~ Day + Sex* + *(1|Subject ID) ‘)*. The results showed that the day had a significant effect, and the CR onset variation tended to increase over 3 days (*t* = 3.04, *p* = 0.002, CI = 2.554, 11.886). Sex did not have significant effect on CR onset latency (*p* = 0.98942) or CR percentage (*p* = 0.96203).

## Discussion

Our study showcases that (1) humans started acquiring embedded eyeblink CR on the first day of training, (2) the CR percentage continued to significantly improve with more days of training, (3) the blink amplitude and frequency improved on days two and three, (4) the blink onset varied over days, and (5) gender did not affect eyeblink CR. This study highlights a significant enhancement in eyeblink conditioning performance across three non-consecutive days of acquisition training, with marked improvements observed on days 2 and 3. This learning trajectory underscores how multiple-day protocols can significantly enhance the CS-US association, thereby strengthening the learning. It also suggests that single-day studies with fewer than 100 trials may overlook the underlying mechanisms of cerebellar consolidation and the benefits of extended training across multiple days.

Because humans can acquire eyeblink CRs within a day and within 100 trials^7^, only a limited number of previous studies^10,13,29–33^ have investigated what happens when you train humans for multiple days. For example, Solomon et al.^10^ reported that multiple day delay eyeblink acquisition improved over 4 non-consecutive days of training in older participants with Alzheimer’s disease. They observed that the eyeblink acquisition was initially lower in participants with AD on day 1 but reached the same levels as the control participants by the end of day 4. In 1996, Woodruff-Pak, Romano & Papka studied older adults with Alzheimer’s disease and Down’s syndrome and age matched controls across 5 consecutive days of eyeblink conditioning study with both delay and trace protocols. They found that all participant groups showed improvement over 5 days with the control group having highest CR percentage^29^. Another study by Woodruff Pak & Finkbiner (1995) involved training younger and older adults for 2 consecutive days. The participants completed ~90 paired trials per day across 2 ISI conditions of 400 ms and 750 ms in a delay paradigm. The researchers reported that learning on day 2 was better and more stable compared to day 1, though not quite statistically significant (*p* = 0.052)^30^. More recent studies by Konrad et al.^31,32^ employed a 3-day non-consecutive delay conditioning on participants ranging from infants to adults to investigate age related changes in learning. They employed 36 paired trials on day 1, ~12 paired trials on day 2 at an ISI of 650 ms and only CS-alone trials on day 3. They reported that the second day of training was necessary to reliably establish learning. Specifically, the low but increasing acquisition rates observed on day 1 significantly increased and peaked on day 2. Yet another 2-day non-consecutive study by Herbert et al.^13^ administered ~38 paired trials in delay paradigm each on 2 days at an ISI of 650 ms in 5-month-old infants and young adults. Their results indicated that although CRs were present on day 1, the CR percentage significantly peaked on day 2 in both the participant groups. They also compared a long-delay protocol and a trace protocol both at an ISI of 1250 ms. They observed that at long delays, the CRs in adults increased and peaked on the second day, while the infants required an additional conditioning session to marginally improve learning. Recently, a smartphone-mediated cerebellar testing platform employed a multiple day delay eyeblink conditioning protocol^33^. The US was a full screen retina flash, and the CS was a white circular dot on the screen. This 14-day study included a total of 6 training sessions with ~50 trials per session at an ISI of 400 ms and reported a gradual increase in the learning across the sessions. In short, most of the previous multiple day eyeblink conditioning studies employed delay or trace protocols and highlighted the influence of participant age and ISI on CR acquisition rates. Their findings indicated that learning strengthened on additional training days. Our 3-day acquisition training spanned over a month, used an embedded protocol and focused on young human adults. We administered 100 paired trials per day at an ISI of 500 ms, and our findings show that acquisition continues into day 2 and day 3 in young human adults with no reported neurocognitive difficulties.

The CS-US relationship and progression of eyeblink conditioning in humans are influenced by the structure of the training protocol. Animals have also shown variations in acquisition rates as the conditioning protocol varies. For example, rigorous training protocols, such as increasing the number of training days or training trials per session or altering the ISI significantly improved delay CR acquisition in rabbits^34^ and in mice^8^. Overall, as the temporal features of the conditioning variables vary, the associated neural mechanisms can vary, which may reflect as variations in the acquisition rate, CR timing or amplitude. Single-day eyeblink conditioning studies demonstrated that reliable levels of CR can be obtained in a day^12,26,35^. Using additional training days, this CS-US association can be further strengthened after day one, with delay, trace or embedded protocols. The cerebellum is key to understanding the mechanisms behind this finding. The cerebellar cortex modulates the amplitude and temporal features of CR^3,36,37^ and strong cerebellar cortex activation has been reported during early and late training trials^12^. The CS-US memory trace is hypothesized to be formed and stored in the deep cerebellar nuclei (DCN), especially the interpositus nucleus (IPN)^3,37,38^. CR consolidation may be mediated by long term depression in the parallel fiber-Purkinje cell synapses or the mossy fiber-deep cerebellar nuclei synapses^39^, or via the cerebellar cortex-interpositus nucleus interactions^37^ or through the cortico-nuclear-olivary feedback loop^40^. However, the exact mechanisms of cerebellar consolidation and memory storage during multiple day eyeblink conditioning are still not very clear to us. We cannot say for certain if a multiple-day paradigm taps solely into the same neural mechanisms as the standard single day eyeblink conditioning paradigm.

With variations in the conditioning protocol, the underlying cerebellar mechanism and the CR acquisition rate vary. For example, the neural circuitry underlying the trace eyeblink conditioning protocol is slightly different owing to the additional reliance on the hippocampal-forebrain circuitry to potentiate the trace interval^2,3,6,7,11,12^. We reported improvement in learning across training days with embedded protocol. However, not much is known about the neurocognitive mechanisms underlying embedded eyeblink conditioning, despite its robustness to the interfering intertrial USs compared to the delay or trace protocols^14,17^. During the embedded protocol, the CS and the US overlap as in the delay protocol, but they do not co-terminate as in the trace protocol. Up until the onset of the US, an embedded protocol is identical to a delay protocol, and since we have focused on changes that occur in this time window, we expect that we would have gotten the same results with a strict delay conditioning paradigm. However, we cannot exclude that the hippocampus or other extracerebellar regions are recruited during the embedded protocol. More research is needed to determine if there are any differences between the protocols in terms of the underlying neural mechanisms.

Eyeblink conditioning is a simple form of cerebellar dependent motor learning^3,41^. Human motor skill acquisition, like learning to ride a bike or playing a musical instrument, is more complex and often not restricted to a single day^42–44^. That is, repetitive practice over days and months, consolidation during sleep and reminiscence improve accuracy required to perform such complex and sequential motor response patterns. However, during eyeblink conditioning, learning is limited by the maximum attainable CS-US associative strength, after which the learning saturates in terms of CR percentage, amplitude and onset^6^. Thus, learning in simple eyeblink conditioning may continue for days until the maximum associative strength is attained, after which paired trials yield saturated CRs reflecting this maximum attainable CS-US associative strength. Eyeblink conditioning can be regarded as a simple motor learning task and various multiple day protocols can be employed to study long-term versus short-term circuit level cerebellar mechanisms^3,45^ of motor learning^46–48^.

An indispensable dialog pertains to factors, such as gender, stress and age, that influence the CR acquisition rate. Eyeblink conditioning is influenced by exposure to stress^26,49^ and normal aging^13^. The rate of acquisition is usually greater among younger adults than older adults^35^. Furthermore, while our study did not identify significant gender differences, aligning with previous literature^50^, young female subjects have shown an increased learning compared to male subjects in humans^51^ and in mice^9,52^. While male and female rats, at any stage of the estrous cycle, were reported to have comparable neurophysiological and behavioral measures^53^, it has also been suggested that the hormone estradiol increases the parallel fiber to Purkinje cell synapses, enhancing cerebellar learning in female mice^54^. Additional data, such as a non-associative control condition or additional training days to establish a learning asymptote, would be beneficial to this literature. Importantly, further research is essential to identify the neurocognitive mechanisms underlying an embedded protocol as well as to compare it with delay and trace protocols.

In summary, most of the previous human studies on delay or trace eyeblink conditioning employed single-day training protocols with the number of trials ranging from 30 to 150. These studies demonstrate that humans can acquire conditioned responses within a day, but they may overlook the underlying mechanisms and benefits of extended training across multiple days. To our knowledge, our study is the first to focus on an embedded protocol for eyeblink conditioning in young human adults across three non-consecutive days of acquisition training. We observed that the learning continues to improve on days two and three, allowing for insights into the effects of extended exposure to eyeblink conditioning. Further investigation is essential to unravel the mechanisms driving cerebellar consolidation and learning in multiple-day embedded eyeblink conditioning. Our findings also call for future studies to compare different training intervals, for example, acquisition trainings that spans over two weeks versus two months. By integrating neuroimaging, electrophysiology, and genetic tools, subsequent research can dissect the cellular substrates of long-term retention and explore how cerebellar-cortical networks adapt across successive training sessions. Additionally, this study can inspire as well as complement future investigations on how the rates of extinction and reacquisition vary over several days of eyeblink conditioning in humans. Such studies will not only refine our understanding of motor learning but may also offer translational insights into cerebellar dysfunction and rehabilitation.

## Methods and materials

### Participants

A total of 45 participants, 26 male and 19 female, with a mean age of 20.92 ± 5.21 years took part in the 3-day eyeblink conditioning experiment. The intervals between training days were not fixed and varied between participants. The mean interval between training sessions one and two was 5 days and the mean interval between training sessions two and three was 7 days. Informed consent was acquired from all participants, who were briefed on the procedures and potential risks prior to participation. Ethical approval was obtained from the Swedish Ethical Review Authority in Lund, Sweden (DNR. 2017/745).

### Experimental setup

Before each training session, participants were equipped with custom-made glasses that had a nozzle for aiming and delivering the air puff, and a GMR chip (AAH002-02E, NVE Corporation) attached to the frame of the glasses. A small circular neodymium magnet (diameter: 3 mm; thickness: 1 mm) was attached to their left eyelid using eyebrow glue. Changes in the magnetic field were recorded using the GMR chip. The GMR sensor data was sampled at 1000 Hz and transferred to a computer via a Micro1401 AD converter (Cambridge Electronic Design). The Micro 1401 was also used to trigger the loudspeakers playing the tone and the delivery of the air puff via triggering a MPPI-3 pressure injector (ASI). Before each session, we tested that the tone was audible to the participant and that the air puff elicited an eyeblink reflex. Participants were allowed to either read a book (many students were preparing for exams), work on an essay or thesis, or watch a program on the computer. If they chose to watch something, they were required to select a program with a low sound profile to minimize auditory interference with the conditioned stimulus (CS). This means that the distractor task varied across participants, introducing a potential confounding variable. However, we chose this approach deliberately: requiring all participants to perform the same task could have led to unequal engagement—some participants might find it stimulating, while others might be bored—which would introduce a different type of confound related to motivation and attention. They were also asked to try not to control their eyelids. They were also asked to try not to control their eyelids.

In our study, an eyeblink was considered as a valid CR if (i) the blink response has an amplitude greater than 25% of the blink amplitude elicited by US and (ii) the blink onset is before the US onset and atleast 100 ms after CS onset. As mentioned before, the emergence of valid eyeblink CRs, measured as a change in the percentage of CRs, can be used to track whether learning takes place or not. The CR percentage was defined in our study as the percentage of trials with valid conditioned eyeblink responses. The CR onset latency was the time elapsed (in ms) between CS onset and CR onset.

### Experimental protocol

**Fig. 4:**
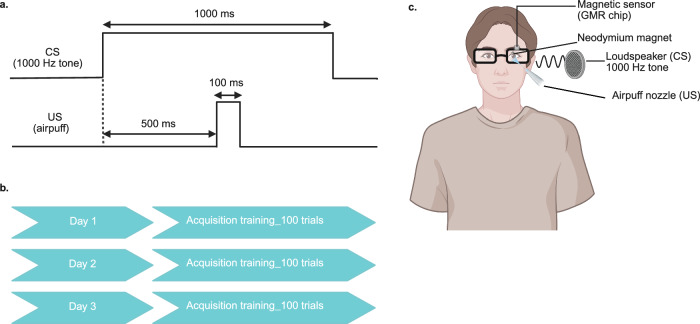
Eyeblink conditioning protocol and experimental setup. **a** A CS-US paired trial using an embedded protocol. **b** 3-day acquisition protocol. **c** The experimental setup consists of custom-made glasses, attached with magnetic sensor and airpuff nozzle. The magnet is glued to the participant’s left eyelid. The CS, US, and GMR chip readings are recorded and controlled via Spike2 v9.10 and CED Micro1401 AD converter. Created in BioRender.com^55^.

A 3-day training protocol was designed to extend beyond the traditional single-day protocols typically employed in human studies and to allow repeated exposure and consolidation of CRs. This approach aimed at exploring whether longer training periods enhance learning compared to single-day protocols. Overall, the 3-day acquisition training consisted of 100 trials per day, resulting in a total of 300 trials. The acquisition training was carried out on 3 non-consecutive days within a month. For the CS, we used a loudspeaker and ensured that the tone was clearly audible while not eliciting any startle response. The CS was a 1000 Hz tone with a 1000 ms duration. The US was an air puff aimed at the left cornea, and the US was embedded within the CS (an embedded protocol). The airpuff intensity varied between 0.5 and 1.5 bar. For each participant, it was adjusted so that it caused a clear blink reflex without feeling aversive. The interstimulus interval (ISI) was 500 ms, and the intertrial interval was 10 ± 2 s. Each participant received 100 trials per day with ~20% randomly interspersed CS-alone trials. The experimental setup and the conditioning protocol are illustrated in Fig. 4.

## Data Availability

The datasets generated and analyzed during this study are available from the corresponding author on reasonable request.

## Code availability

The codes used in this study are available from the corresponding author upon reasonable request.
